# Methylation status of hypothalamic *Mkrn3* promoter across puberty

**DOI:** 10.3389/fendo.2022.1075341

**Published:** 2023-01-13

**Authors:** Pavlos Fanis, Maria Morrou, Marios Tomazou, Kyriaki Michailidou, George M. Spyrou, Meropi Toumba, Nicos Skordis, Vassos Neocleous, Leonidas A. Phylactou

**Affiliations:** ^1^ Department of Molecular Genetics, Function and Therapy, The Cyprus Institute of Neurology and Genetics, Nicosia, Cyprus; ^2^ Department of Bioinformatics, The Cyprus Institute of Neurology and Genetics, Nicosia, Cyprus; ^3^ Biostatistics Unit, The Cyprus Institute of Neurology and Genetics, Nicosia, Cyprus; ^4^ Child Endocrine Care, Department of Pediatrics, Aretaeio Hospital, Nicosia, Cyprus; ^5^ Division of Pediatric Endocrinology, Paedi Center for Specialized Pediatrics, Nicosia, Cyprus; ^6^ Medical School, University of Nicosia, Nicosia, Cyprus

**Keywords:** DNA methylation, MKRN3, puberty timing, CpG island, promoter

## Abstract

Makorin RING finger protein 3 (MKRN3) is an important factor located on chromosome 15 in the imprinting region associated with Prader-Willi syndrome. Imprinted *MKRN3* is expressed in hypothalamic regions essential for the onset of puberty and mutations in the gene have been found in patients with central precocious puberty. The pubertal process is largely controlled by epigenetic mechanisms that include, among other things, DNA methylation at CpG dinucleotides of puberty-related genes. In the present study, we investigated the methylation status of the *Mkrn3* promoter in the hypothalamus of the female mouse before, during and after puberty. Initially, we mapped the 32 CpG dinucleotides in the promoter, the 5’UTR and the first 50 nucleotides of the coding region of the *Mkrn3* gene. Moreover, we identified a short CpG island region (CpG islet) located within the promoter. Methylation analysis using bisulfite sequencing revealed that CpG dinucleotides were methylated regardless of developmental stage, with the lowest levels of methylation being found within the CpG islet region. In addition, the CpG islet region showed significantly lower methylation levels at the pre-pubertal stage when compared with the pubertal or post-pubertal stage. Finally, *in silico* analysis of transcription factor binding sites on the *Mkrn3* CpG islet identified the recruitment of 29 transcriptional regulators of which 14 were transcriptional repressors. Our findings demonstrate the characterization and differential methylation of the CpG dinucleotides located in the *Mkrn3* promoter that could influence the transcriptional activity in pre-pubertal compared to pubertal or post-pubertal period. Further studies are needed to clarify the possible mechanisms and effects of differential methylation of the *Mkrn3* promoter.

## Introduction

Puberty is the transition period between childhood and adulthood required in mammals to develop secondary sexual characteristics and become reproductive functionally (1). The range and onset of puberty are altered by complex interactions between genetic, environmental and nutritional factors (2). The hypothalamic-pituitary–gonadal (HPG) axis regulates the initiation of puberty through an increase in pulsatile production of gonadotropin-releasing hormone (GnRH) followed by a sequence of neuroendocrine events (3, 4). In humans, changes in the onset of puberty result in a variety of clinical conditions such as precocious and delayed puberty (5). Early maturation of the entire HPG axis results in gonadotropin-dependent precocious puberty which is characterized by increased expression of GnRH in the hypothalamus and the development of secondary sexual characteristics before the age of 8 years in girls and 9 years in boys (3, 4, 6). Globally, the prevalence of CPP is estimated between 0.01 – 0.43% (7–10). To date, mutations in the *KISS1*, *KISS1R*, *PROKR2*, *DLK1* and the *MKRN3* genes were identified as causative for CPP with *MKRN3* defects being the most frequent genetic cause reported (11–22).

*MKRN3* is an intronless gene that belongs to the *MKRN* gene family with a high level of conservation in mammals (23) with the mouse *Mkrn3* ortholog being 82% identical to the human *MKRN3* (24). Four functional *MKRN* genes *MKRN1*, *MKRN2*, *MKRN3* and *MKRN4* have been described in the literature (23). MKRN3 is a 507 amino acid protein consisting of five zinc finger domains: three C3H motifs in the N-terminal region, one C3HC4 RING motif and one MKRN-specific Cys-His domain (25). Loss of function mutations in the *MKRN3* gene results in premature activation of the HPG axis by activating GnRH secretion. To date, more than 50 mutations have been mainly identified in the coding region of the gene (13, 16–22). Nevertheless, few studies describe causative alterations occurring in the 5’UTR and promoter regulatory regions of the *MKRN3* gene (26–28). The specific mechanism of MKRN3 repression activity is still under investigation, however, recent studies attempt to elucidate it. In female rats, binding of mir-30b in the 3’ UTR of *Mkrn3* suppresses the function of the Mkrn3 repressor, thus regulating the onset of puberty (29). In addition, MKRN3 represses the promoter’s transcription activity of two key pubertal factors, *KISS1* and *TAC3*; this activity may implicate an ubiquitin-mediated mechanism controlled by MKRN3 (30). MKRN3 ubiquitinates various factors in order to suppress GNRH expression. Ubiquitination of MBD3 transcriptional repressor inhibits both it’s binding to the GNRH1 promoter and the DNA TET2 demethylase resulting in epigenetically suppression of GNRH1 transcription (31). Moreover, MKRN3-mediated ubiquitination of the poly(A)-binding proteins PABPC1, PABPC3, and PABPC4, reduce their binding to the poly(A) tail of target mRNAs, including GNRH1 mRNA, affecting their stability and translation (32).

Human *MKRN3*, is located on chromosome 15q11-q13 within the critical region of Prader-Willi syndrome (PWS) (25). The mouse *Mkrn3* ortholog is located in the chromosome 7C region (33). In both species, *MKRN3* is maternally imprinted and paternally expressed with imprinting function to be controlled by an imprinting control region (ICR) upstream of the *SNURF/SNRPN* gene (34, 35). Imprinted genes play important roles in pre- and postnatal development. In humans, abnormal imprinting is responsible for certain congenital disorders (36), while disturbed imprinting is associated with cancers (37). Most imprinted genes are found in clusters, forming imprinting regions with genes expressed paternally or maternally. The monoallelic expression of the imprinted genes is regulated by differentially methylated regions (DMRs) inherited from germline DMRs. A DMR is present at the imprinted locus and includes a region rich in CpG dinucleotides called the imprinting control region (ICR). ICRs control the imprinted genes through a monoallelic DNA methylation status and are located in either intergenic or promoter regions (38, 39). Additional DMRs are subsequently obtained after implantation phases in response to the germline DMRs, which are also necessary for the imprinted expression of a specific gene in the cluster (38). In the human PWS imprinted region there are three maternally methylated DMRs, one germline DMR of the PWS imprinting center (PWS-IC) and two somatic DMRs at the *NDN* and *MKRN3* genes (25, 40, 41).

The process of puberty is largely controlled by epigenetic mechanisms that include, among other things, DNA methylation of puberty-related genes (42). DNA methylation of CpG dinucleotides is an important epigenetic event that regulates the expression of mammalian genes in which, through the process of differentiation, different cell types develop their specific methylation profile. RNA sequencing together with whole genome methylation studies in prepubertal and pubertal goat hypothalamus have shown that DNA methylation regulates the onset of puberty (43). Differential methylation was observed in several non-coding regions, including promoters, UTRs and introns (43). In cultured rhesus monkey GnRH neurons during GnRH neuronal development, an increase in GnRH gene expression and a decrease in CpG methylation status was observed. These findings suggest that demethylation of GnRH CpGs eliminates suppression on GnRH gene transcription (44). During the pre-pubertal period, in the hypothalamus, the expression of genes that are activated during puberty is suppressed (45, 46). Such is the case with the *KISS1* gene, the expression of it is repressed by methylation in the CpG islands of its promoter region and consequently recruiting the Polycomb repressor complex (46, 47).

Here, we report the comprehensive methylation status of the promoter, the 5’UTR and the first 50 nucleotides of the coding region of the *Mkrn3* gene in three developmental stages, before, during and after puberty. We defined a short CpG island (CpG islet) consisting of seven CpG dinucleotides that is differentially methylated before and after puberty and performed *in silico* analysis for the binding of potential transcriptional regulators.

## Materials and methods

### Experimental design

**Figure 1:**
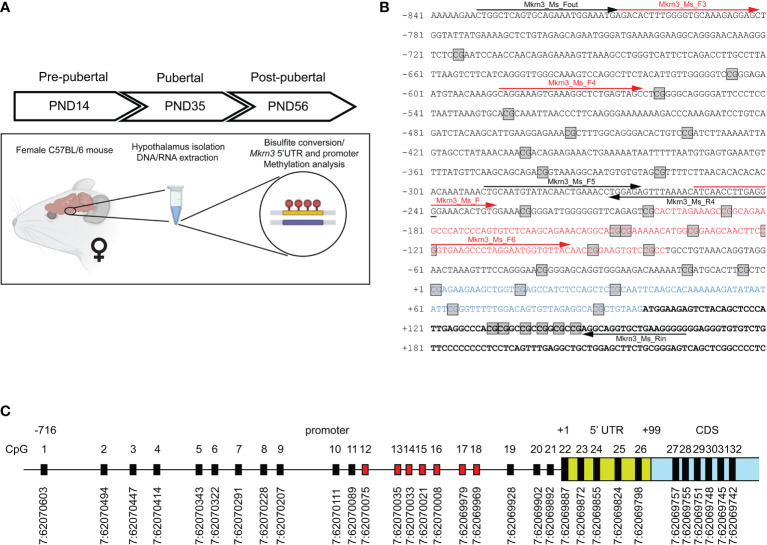
Mapping of CpG dinucleotides in the promoter of *Mkrn3.* **(A)** Experimental design of the study. **(B)** Nucleotide sequence of *Mkrn3* promoter, 5’UTR and beginning of the coding region under study. PCR primers are indicated with black arrows. Sequencing primers are indicated with red arrows. Nucleotide sequence of the CpG islet is indicated with red color. Nucleotide sequence of the 5’UTR is indicated with blue color. CpG dinucleotides are depicted in square boxes. **(C)** Schematic representation of the CpG dinucleotides in the *Mkrn3* region under study with chromosomal locations according to GRCm39/mm39. CpG dinucleotides of the CpG islet are indicated with red color. CDS, Coding DNA Sequence.

Hypothalamic tissue of female mice was isolated in three stages Postnatal Day 14 (PND14), Postnatal Day 35 (PND35) and Postnatal Day 56 (PND56) corresponding to developmental stages before, during and after puberty respectively. We initially map CpG dinucleotides to a region 850bp upstream and 150bp downstream of the transcription start site (TSS) of the *Mkrn3* gene. The upstream promoter region was selected based on data from the ReMap tool (48), chr7:62,069,224-62,071,878, which are integrated and presented with the UCSC Genome Browser on Mouse (GRCm39/mm39) https://genome.ucsc.edu (**Supplementary Figure 1**). Genomic DNA was extracted, bisulfite converted, followed by methylation-specific PCR and sequencing of the *Mkrn3* promoter, 5’UTR and the first 50 nucleotides of the coding region. The methylation status of the individual CpG sites was examined by statistical and *in silico* tools (**Figure 1A**).

### Animals and tissue isolation

C57BL/6 mice were housed in open-top cages in a controlled temperature, humidity and pathogen-free mouse facility. Three female mice for each developmental stage of PND14, PND35 and PND56 were used in this study. The day the mice were born was listed as PND1 of age. The PND35 and PND56 mice were weaned at PND21 and kept in a 12 hrs dark/12 hrs light cycle at a temperature of 22°C with access to water and standard diet pelleted food *ad libitum*. Female C57BL/6 mice at the stated ages (PND14, PND35, PND56) were sacrificed by cervical dislocation, in the morning (09:00 – 11:00 AM). Prior of sacrificing, vaginal opening and estrus cycle stage, using the smear method (49), were determined. The vaginal opening was detected in PND35 and PND56 mice but not in PND14 mice. For this study, PND35 and PND56 mice that were on metestrus stage of estrus cycle were sacrificed. In our mouse facility, vaginal opening on C57BL/6 mice is observed at an average of 28 days. Brains were removed and whole hypothalamus were dissected, immediately frozen in liquid nitrogen and stored at -80°C. All experimental procedures in this study were performed in accordance with animal care protocols approved by the Cyprus Government’s Veterinary Services (project license CY/EXP/PR.L4/2018) according to national law, which is harmonized with EU guidelines (EC Directive 86/609/EEC).

### DNA/RNA preparation

The whole hypothalamic tissue was thawed at room temperature and homogenized in 500 μl of TRIzol reagent (Invitrogen, Carlsbad, CA, USA). RNA and genomic DNA was isolated according to the TRIzol reagent manufacturer instructions. The yield of RNA and DNA was determined using the NanoDrop One spectrophotometer (ThermoFisher Scientific, Waltham, MA, USA).

### Bisulfite sequencing

Methylation-specific primers were designed using the EpiDesigner website tool (http://www.epidesigner.com/) (Agena Bioscience, Hamburg, Germany) targeting the mouse *Mkrn3* 5’ UTR and promoter region (**Figure 1B**). 350 ng of genomic DNA were sodium bisulfite treated using the EpiJET Bisulfite Conversion Kit according to manufacturer instructions (Thermo Scientific, Pittsburg, PA, USA). Briefly, DNA denaturation and sodium bisulfite conversion reaction were performed in one-step according to Protocol A of the kit for higher sensitivity. A volume of 20μl of DNA was mixed with 120μl of prepared Modification Reagent and proceeded to a reaction with initial denaturation temperature at 98°C for 10min, followed by bisulfite conversion at 60°C for 150min. This step included the deamination of all unmethylated cytosines to uracils while methylated cytosines remained unchanged, to be detected as thymines in the following PCR applications. After DNA conversion, a desulphonation and DNA purification step was performed, using DNA Purification Micro Columns. All centrifugation steps were carried out at 12.000 rpm for 30-60sec and the flow-through was discarded each time. Converted DNA sample was loaded in the column with 400μl of prepared Binding Buffer A and proceeded to centrifugation. A washing step of the micro column with the prepared Wash Buffer was performed, followed by the addition of 200μl of Desulphonation Buffer, prepared with ethanol. Column was left at room temperature for 20min, followed by centrifugation. After two sequential washing steps, the purified converted DNA was eluted using 20μl of the provided Elution Buffer.

PCR amplification was performed using the bisulfite treated DNA as template. To optimize the reaction, different sets of primers were designed and tested, as well as different Taq DNA polymerases and PCR cycle conditions. For the optimal result, the region of interest was amplified in two separated PCR reactions using the following primers; PCR1: Mkrn3_Ms_Fout: 5′-TTGGTTTAGTGTAGAAATGGAAATGA−3′, Mkrn3_Ms_R4: 5′-CCCTCAAAATTAATATTTTAAACTCTCC−3′; PCR2: Mkrn3_Ms_F5: 5′-TTGTAATGTATATAATTGAAATTTGGAGA−3′, Mkrn3_Ms_Rin: 5′-CCCCTTCAACACCTACCT−3′. PCR reactions consisted of 2 μl PCR Buffer (10x), 2 μl dNTPs (2mM), 1 μl of each primer (10μM), 0.8 μl MgCl_2_ (25mM), 0.2 μl HotStarTaq^®^ Plus DNA Polymerase (Qiagen, Hilden, Germany), 2 μl bisulfite treated DNA at a final volume of 20 μl. Amplification was performed with an initial denaturing temperature at 95°C for 5min, followed by 45 cycles of denaturation (95°C, 45sec), annealing (56°C, 1min), extension (72°C, 1min), with a final extension at 72°C for 10min. PCR products were cleaned-up with ExoSAP-IT reagent (Applied Biosystems, Foster City, CA) and sequenced using the BigDye™ Terminator v1.1 Cycle Sequencing Kit (Applied Biosystems, Foster City, CA) with the following internal primers; PCR1: Mkrn3_Ms_F3: 5′-GATATTTTGGGGTGTAAAGAGGAGT−3′, Mkrn3_Ms_F4: 5′- TAGGAAAGTGAAAGGTTTTGAGTAGT−3′; PCR2: Mkrn3_Ms_F: 5′-ATTAATTTTGAGGGGAAATATTGTG−3′, Mkrn3_Ms_F6: 5′-GGTGAAGTTTTAGGAATGGTGTTAT−3′ on a 3500xL Genetic Analyzer (Applied Biosystems, Foster City, CA) (**Figure 1B**).

### Bisulfite sequencing analysis

Sanger sequencing files (.abi format) were analysed in R (50) using the sangerseqR (51) package for reading, parsing, base calling and obtaining the matrices of intensity traces and their corresponding peaks. The primary, secondary, reference and converted reference sequences were processed using the Biostrings (52) package and aligned using the AlignSeqs function from the DECIPHER (53) package. Following the alignment, the C/T peak intensity ratios at the C positions of the reference sequence were calculated and then filtered to identify cytosines within a CpG context (C followed by a G). The results were combined by group and exported in.csv format for further processing.

### RT-qPCR

Prior to cDNA preparation 500ng of RNA was treated with DNaseI (Invitrogen, Carlsbad, CA, USA). cDNA synthesis reaction initially consisted of 500ng of DNaseI treated RNA, 2 μl d(T)23VN (50 µM), 1 μl dNTPs (10mM) in a final volume of 10 μl. The reaction was incubated at 65°C for 5 min and kept on ice and the following components added: 2 μl M-MuLV buffer (10X), 1 μl M-MuLV RT (200 U/µl), 0.2 μl RNase Inhibitor (40 U/µl) in a final volume of 20 μl. The cDNA synthesis reaction was incubated at 42°C for 1 hour followed by incubation at 65°C for 20min. All components of cDNA synthesis reaction were from New England Biolabs, Ipswich, MA, USA. For qPCR, the cDNA product was diluted four times and 2 μl were used. qPCR reaction consisted of 2 μl cDNA, 5 μl SYBR™ Green PCR Master Mix (2x) (Applied Biosystems, Foster City, CA), 0.2 μl of each primer (10 µM) in a final volume of 10 μl and performed on the QuantStudio™ 3 Real-Time PCR System (Applied Biosystems, Foster City, CA). Each sample was amplified in triplicate. Relative expression analysis was performed as described before (54). Cycle threshold levels were calculated for each gene and normalized to values acquired for the endogenous Actin house-keeping gene. The following primers were used for qPCR experiments: Actin_RT_F: 5′-GCTTCTTTGCAGCTCCTTCGT-3′, Actin_RT_R: 5′-CCAGCGCAGCGATATCG-3′, Mkrn3_RT_F: 5′-TCCTGGACAGCCTTACCG-3′, Mkrn3_RT_R: 5′-TATGCACACCTGTCCCCAC-3′.

### CpG island prediction

CpG islands in *Mkrn3* promoter sequence were predicted using EMBOSS Cpgplot (55). EMBOSS Cpgplot predicts potential CpG islands in the input DNA sequence. The size of the input sequence was 1080bp covering 841bp upstream of the TSS and 240bp downstream of the TSS; mouse genome assembly: GRCm39/mm39 (GCA_000001635.9), positions 7:62070722 – 7:62069604. The parameters used for CpG island prediction were; Window Size: 100bp, Minimum length of an island: 200 and/or 100bp, Minimum observed/expected no of CpG dinucleotides: 0.6, Minimum average percentage of GC: 50.

### *In silico* transcription factor binding site analysis

*In silico* prediction and analysis of transcription factor binding sites at the Mkrn3 CpG islet region between -80bp to -200bp from TSS were performed using the CiiiDER tool (56). The parameters used were; the default deficit cut-off at 0.15, that means the scan will accept any transcription factor binding sites that have MATCH scores of 0.85 or above and the JASPAR 2020 CORE collection matrix for Mouse.GRCm38.94. Only transcription regulators predicted in the positive DNA strand were selected for further analysis. The predicted transcription regulators were crosschecked using the Transcription Factor Binding Site Prediction function of AnimalTFDB4 database (57).

Hypothalamic RNA sequencing data for the predicted transcription regulators were extracted from the Brain Atlas resource [part of the Human Protein Atlas (58)]. RNA sequencing data were derived from two female and two male mice at PND56 and expression levels presented as normalized transcripts per million (nTPM).

### Statistical analysis

For the comparison of the methylation at each CpG site at the three different developmental stages, we performed pairwise t-tests. The comparison of the CpG sites as grouped into regions were performed using repeated measures of ANOVA followed by post-hoc tests. Post-hoc test p-values were adjusted using the Bonferroni correction. For the Mkrn3 expression analysis at the three different developmental stages, we performed one-way analysis of variance (ANOVA) followed by Tukey’s Post-hoc test. All analyses were performed using the statistical software R and all tests were two sided. Adjusted p-values at p<0.05 were considered as significant. Correlations between CpG region methylation across developmental stages and Mkrn3 expression have been calculated using the Pearson’s correlation coefficient.

## Results

### Mapping of CpG dinucleotides in *Mkrn3*

Variations in methylation levels occurring at CpG dinucleotides in mammalian gene promoter regions have been linked to gene regulation. In the present study, we investigated the potential effect of promoter methylation, 5’ UTR and the first 50 nucleotides of the *Mkrn3* coding region across puberty in female mice. Therefore, methylation of DNA derived from the hypothalamic tissue of female mice has been studied in three developmental stages, pre-pubertal (PND14), at puberty (PND35) and post-pubertal (PND56) (**Figure 1A**). Initially, we mapped the CpG dinucleotides located 850bp upstream and 150bp downstream of the TSS. The 850bp promoter region of *Mkrn3* under study has 21 CpG dinucleotides, the 100bp 5’UTR has 5 CpG dinucleotides and the first 150bp of CDS has 6 CpG dinucleotides (**Figures 1B, C**). The density of CpG sites vary across the region under investigation with CpG sites appear to be denser in the region spanning the TSS. For the identification of possible CpG island(s) in the *Mkrn3* promoter, *in silico* analysis was performed using the EMBOSS Cpgplot tool (55). Initial analysis using the default parameters as defined by Gardiner-Garden and Frommer (59), which are a minimum island length: 200bp, a CG : GC ratio equal to or greater than 0.6 and a GC percentage greater than 50% did not yield any results. However, when the minimum island length was set to 100bp, analysis revealed a putative short CpG island with a length of 120bp that extended from -80bp to -200bp upstream of the TSS that contained seven CpG dinucleotides (CpG12-18) (**Figures 1B, C**). Such genomic regions shorter than 200bp but with the same GC content and CpG ratio are recognized as CpG islets (60).

### Methylation status of CpG sites in *Mkrn3*

Since epigenetic changes have been widely associated with puberty, we evaluated the methylation levels of the promoter, the 5’UTR and the first 50 nucleotides of the coding region of the *Mkrn3* gene in the hypothalamus of the female mice at three developmental stages, pre-pubertal (PND14), at puberty (PND35) and post-pubertal (PND56). Of the 32 CpG sites under study, one CpG site (CpG10) had insufficient coverage for analysis.

**Figure 2:**
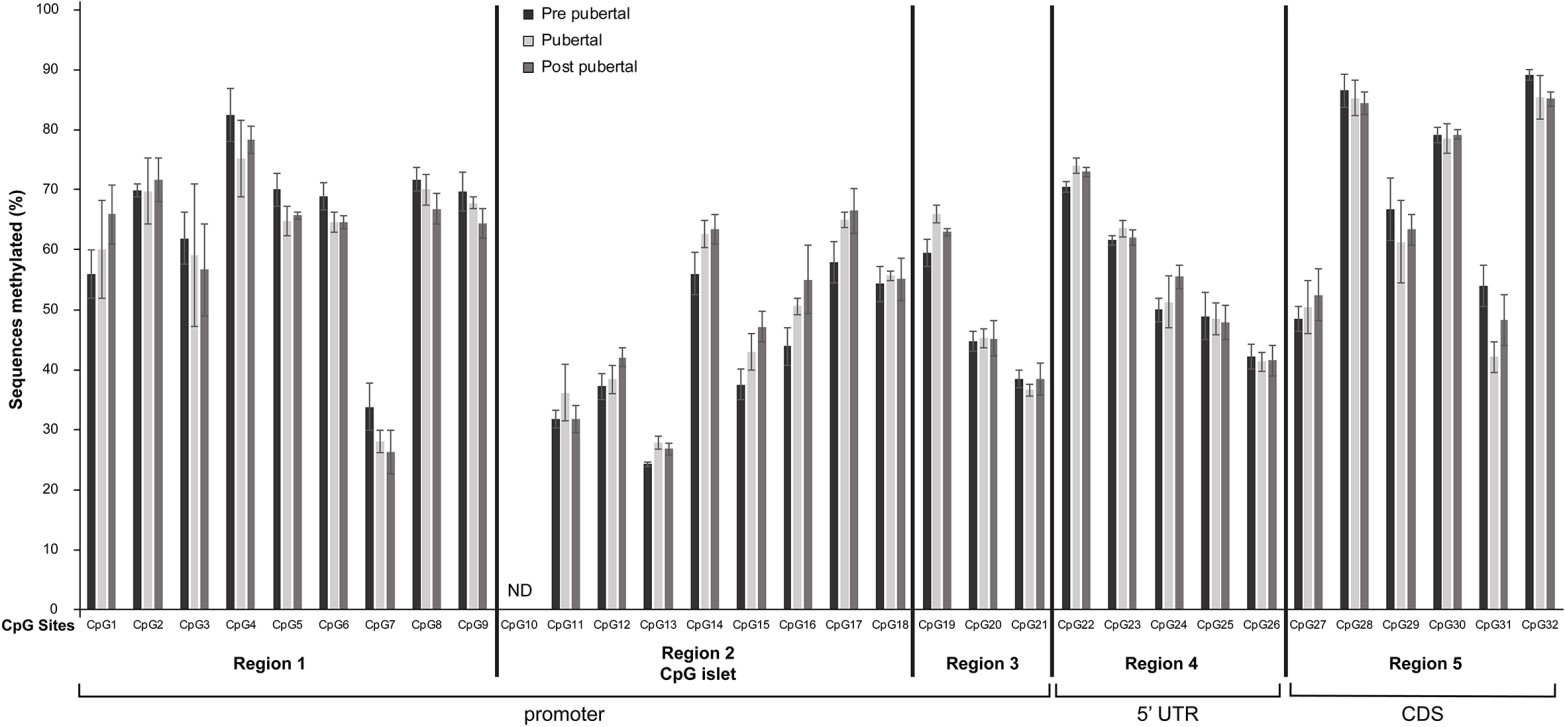
Differential methylation of CpG dinucleotides located in the promoter, 5’UTR and the first 50 nucleotides of the coding region of *Mkrn3* gene. Bar graphs showing the percentage of methylated CpGs in hypothalamus at pre-pubertal, pubertal and post-pubertal developmental stages. The five grouped regions are depicted with black vertical lines. CDS, Coding DNA Sequence; ND, no data.

The CpG dinucleotides where grouped in five regions. Region 1 consists of nine CpG dinucleotides (CpG1 – CpG9) located from -716bp to -321bp upstream of TSS; region 2 consists of nine CpG dinucleotides (CpG10 – CpG18) located in the CpG islet and extending from -225bp to -83bp upstream of TSS; region 3 consists of three CpG dinucleotides (CpG19 – CpG21) and extending from -42bp to -5bp upstream of TSS; region 4 consists of five CpG dinucleotides (CpG22-CpG26) located in the 5’UTR region of the gene and region 5 consists of 6 CpG dinucleotides (CpG27 – CpG32) located in the first 50 nucleotides of the coding region of the gene (**Figure 2**).

Overall, the 31 CpG sites under study were mostly methylated irrespectively of the developmental stage. Specifically, 14 CpG sites, CpG7, CpG11, CpG12, CpG13, CpG15, CpG16, CpG18, CpG20, CpG21, CpG24, CpG25, CpG26, CpG27 and CpG31 showed ≤ 50% methylation levels and the remaining 17 CpG sites were >50% methylated regardless the developmental stage (**Figure 2**). Interestingly, the lower methylation level CpG sites, CpG11, CpG12, CpG13, CpG15, CpG16 and CpG18 located in the CpG islet while the CpG20 and CpG21 located next to the TSS (**Figure 1C**).

None of the Individual CpG dinucleotides showed significantly different methylation at the pre-pubertal stage (PND14) when compared with the pubertal stage (PND35) or at the pre-pubertal stage (PND14) when compared with the post-pubertal stage (PND56) (**Figure 2**).

**Figure 3:**
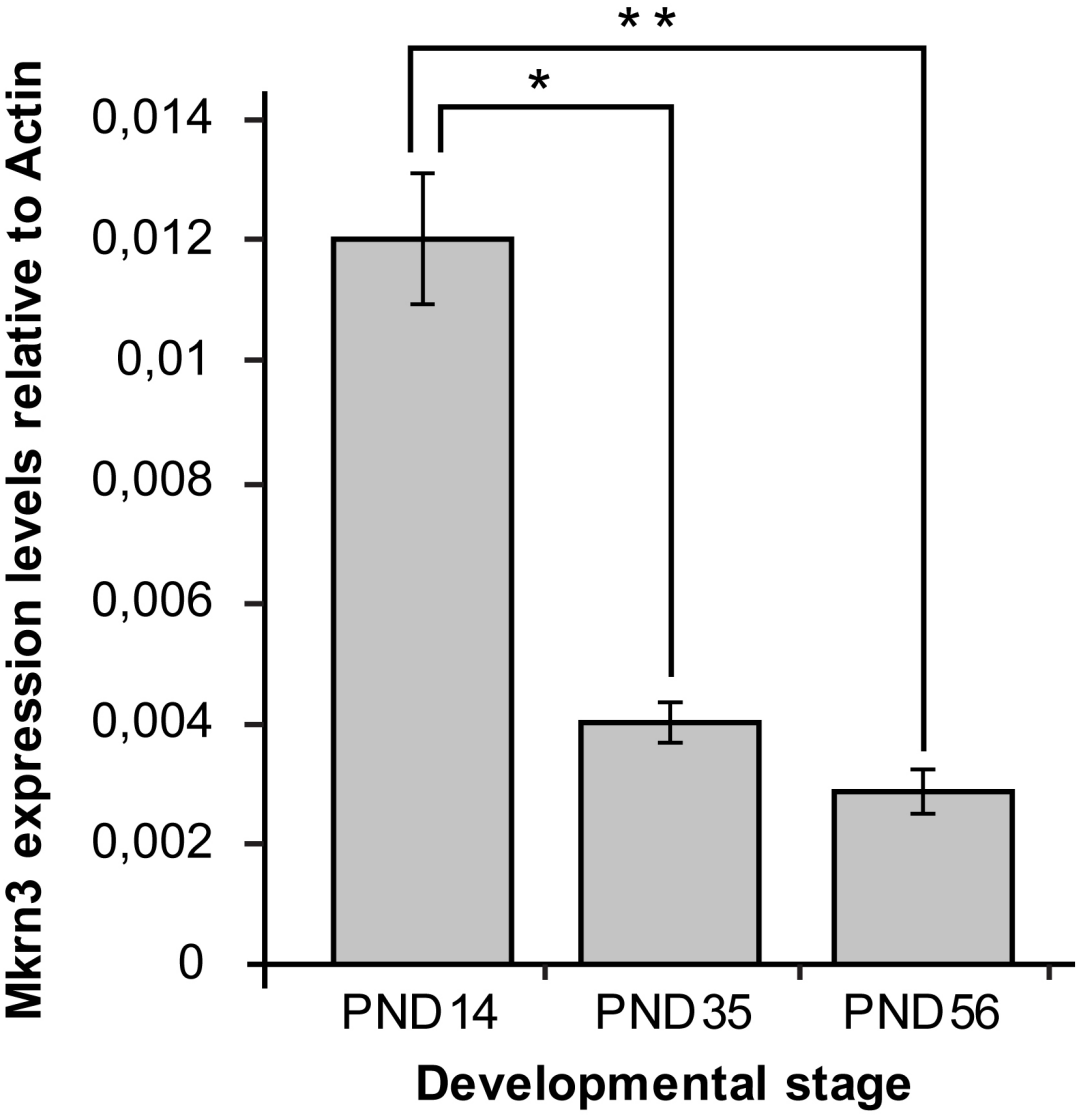
Hypothalamic Mkrn3 mRNA expression at pre-pubertal (PND14), pubertal (PND35) and post-pubertal (PND56) stages. *, P= 0.0000185, **, P= 0.0000082, one-way analysis of variance (ANOVA).

In order to determine the association between *Mkrn3* promoter methylation and gene expression, quantitative PCR was performed for *Mkrn3* in the same hypothalamic samples. Expression analysis showed approximately 70% decrease between prepubertal and pubertal samples, which continues to remain at low levels after puberty (**Figure 3**). These findings were consistent with previous observations (13, 30). Comparison of Mkrn3 expression with individual CpG dinucleotide or overall *Mkrn3* promoter methylation across puberty showed no association. However, methylation differences of CpG dinucleotides located in the CpG islet region where transcription factors may bind can explain some gene activity.

### Differential methylation of *Mkrn3* CpG islet across puberty

**Figure 4:**
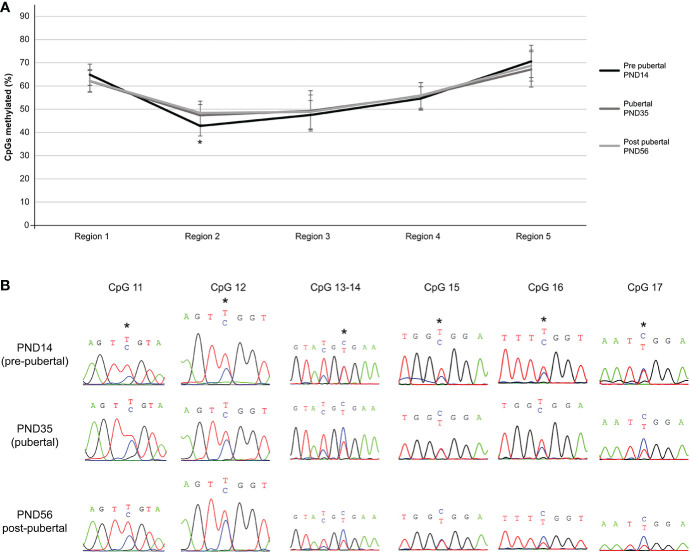
Differential methylation of the five regions of the studied *Mkrn3* locus. **(A)** Line graphs showing the percentage of methylated CpGs in the five regions of hypothalamic *Mkrn3* locus under study at pre-pubertal, pubertal and post-pubertal developmental stages. *, P=0.0009, one-way analysis of variance (ANOVA). **(B)** Sequence electropherograms of CpG sites CpG11, CpG12, CpG13, CpG14, CpG15, CpG16 and CpG17 located at the *Mkrn3* CpG islet at three developmental stages across puberty. Methylated cytosine residue is depicted in blue color. Asterisks showed the differential methylated nucleotides.

Despite non-significant methylation differences of individual CpG dinucleotides of the *Mkrn3* region studied across puberty, there were significant methylation differences when CpG dinucleotides were grouped into regions. Overall, irrespective of the developmental stage, region 2, which contained the CpG islet, showed the lowest methylation levels between 42-48%, followed by region 3 with methylation levels between 47-49%. Regions 1, 4 and 5 showed methylation levels between 62-70%. Notably, only region 2 showed significantly (p value = 0.0009) lower methylation levels by 5% at the pre-pubertal stage (PND14) when compared with the pubertal stage (PND35) (**Figures 4A, B**). The differences in methylation during puberty by 6% were still significant (p value= 0.007) when the pre-pubertal (PND14) was compared with the post-pubertal (PND56) hypothalamus (**Figures 4A, B**). Comparison of CpG region methylation in relation to Mkrn3 expression across stages using the Pearson’s correlation coefficient showed for region 2 the highest value of -0.70 indicating a moderate negative linear relationship. Thus, region 2 of the *Mkrn3* promoter which contains the CpG islet showed differential methylation that could affect transcriptional activity, in the pre-pubertal versus adolescent or post-pubertal period.

### *In silico* analysis of transcription factor binding sites at *Mkrn3* CpG islet

**Figure 5:**
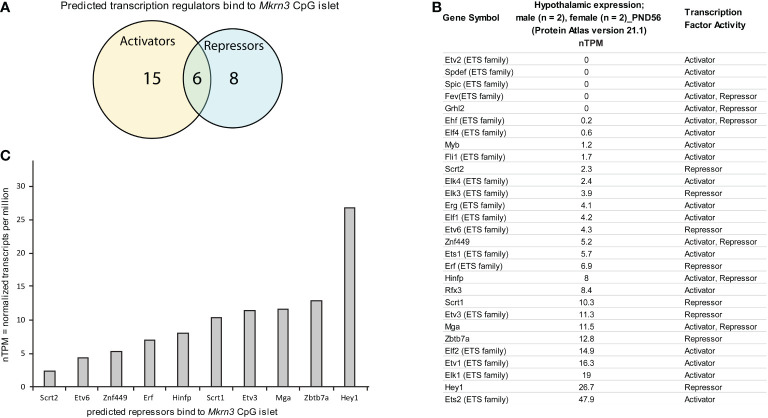
*In silico* analysis of transcription factor binding sites at *Mkrn3* CpG islet. **(A)** Venn diagram showing the predicted transcription activators and repressors bind to *Mkrn3* CpG islet. **(B)** Hypothalamic expression at post-pubertal stage (PND56) of the predicted transcription regulators bound to *Mkrn3* CpG islet. RNA sequencing data of mice (two males and two females) at PND56 were extracted from the Brain Atlas resource [part of the Human Protein Atlas (58)]. **(C)** Bar graphs showing the hypothalamic expression of predicted repressors bind to *Mkrn3* CpG islet at post-pubertal stage (PND56). nTPM: normalized Transcripts Per Million.

Since the majority of the significant low methylated nucleotide residues were located in the CpG islet we further performed an *in silico* mapping for transcription factors that may bind to it. Using the CiiiDER *in silico* tool, the CpG islet region appears to be bound by various transcription factors either with activation or repression capability. Specifically, 29 factors are predicted to have a binding affinity within the CpG islet region between -80bp to -200bp, with 15 factors acting as activators, 8 factors acting as repressors and 6 factors acting both as activators and as repressors (**Figure 5A**). It is worth noting that 19 of the transcription factors belong to the E26 Transformation Specific (ETS) transcription family (61) and since they bind to similar sequence motifs appear in the prediction results (**Figure 5B**). The ETS family consists of both transcriptional activators and repressors. Among other functions, they appear to have a role in the endocrine system, such as controlling the expression of specific genes in the pituitary gland, mammary gland development, and their involvement in breast, prostate, and reproductive organ cancers (62).

In order to evaluate the 29 predicted transcription factors, hypothalamic RNA sequencing data of mice at PND56 were extracted from the Brain Atlas resource [part of the Human Protein Atlas (58)]. Expression levels, presented as nTPM, for each of the transcription factors were variable, with values ranging from zero to 47 nTPM (**Figure 5B**). Due to the fact that the data derived from mice at PND56 (two females and two males), when Mkrn3 is downregulated, we focused on factors that showed repressor activity. Selection analysis resulted in ten repressors with expression levels ranging from 2.3 to 26.7 nTPM (**Figure 5C**). Among the selected predicted repressors, Hinfp with expression levels of 8 nTPM, encodes a transcription factor that interacts with the methyl-binding protein MBD2 and plays a role in DNA methylation and repression (63). In addition, Zbtb7a with expression levels of 12.8 nTPM, encodes a factor that represses the transcription of various genes through interaction with the NuRD repressor complex or by recruiting the chromatin regulator HDAC1 (64). The transcriptional repressor HEY1 with expression levels of 26.7 nTPM is a downstream effector of the Notch signalling pathway (65), a key pathway for kisspeptin neuron development (66). HEY1 is expressed in the anterior pituitary progenitor cells during postnatal development and in the adult pituitary (67). Therefore, the distinctive methylation pattern at the CpG islet of *Mkrn3* promoter across puberty may control the binding of specific transcription regulators.

## Discussion

Loss of function mutations in the intronless *MKRN3* gene have been identified as the most frequent genetic cause of familial non syndromic CPP, due to the premature activation of the hypothalamic-pituitary-gonadal axis (68, 69). *MKRN3* is a maternally imprinted gene located within the critical region of PWS on chromosome 15q11-q13 (25), showing an important role of DNA methylation in the mechanisms of the puberty process. Several studies have examined the imprinting status of the PWS critical region with emphasize only in specific DMRs and locations (70–72). Bessa et al, investigated the methylome profile of healthy and central precocious puberty girls identifying several hypermethylated DMRs with only one of them in the ZFP57 gene region being hypomethylated (73). Due to the inability to obtain a sample of brain tissue, in most cases, peripheral blood leukocytes were used as the primary sample.

In this original study, we showed that a CpG islet in the *Mkrn3* promoter is differentially methylated at the onset of puberty. Here we performed a comprehensive examination of *Mkrn3* promoter and 5’UTR methylation in female mouse hypothalamic tissues during puberty. The *Mkrn3* promoter demonstrated differential DNA methylation in association with reduced Mkrn3 expression coincident with onset of puberty, but it is uncertain whether the alterations in methylation were causative. In support of the important role of the promoter in the regulation of transcription, in humans, mutations in the promoter region of *MKRN3* have been linked to the transcriptional activity of the gene (26–28).

Differential methylation at promoter regions important for transcription is important for the regulation of gene expression. Significant differential promoter methylation of *Kiss1* and *Kiss1r*, two important regulators of pubertal development, was observed across puberty in female rats (74). Increasing evidence points to the involvement of epigenetic mechanisms controlling the hypothalamic onset of puberty. Studies in mice have shown that MBD3, a methyl-CpG-binding protein, is ubiquitinated by MKRN3 resulting in disruption of its binding to the GNRH1 promoter and subsequent silencing of GNRH1 expression (31).

Due to the imprinting status of the PWS region in which *Mkrn3* is located, the changes of methylation at individual CpG dinucleotides are small. However, overall methylation of the CpG islet, consisting of seven CpG sites, showed significantly increased methylation during and after puberty (**Figure 4A**). CpG islands and CpG islets are promoter regions rich in CpG dinucleotides where DNA methylation control gene expression through transcriptional silencing of the corresponding gene (75).

A limitation in elucidating the data of this study is that they are derived from the hypothalamus, a tissue with a mixed cell population consisting, among other cells, of different neurons. It is possible that the pattern and level of specific methylation observed in this study will become clearer when using genomic DNA from distinct neurons such as GnRH and Kiss1 neurons.

*In silico* analysis revealed a number of potential transcriptional regulators with binding affinity to the *Mkrn3* CpG islet. Potential transcriptional regulators were screened for their hypothalamic expression in adult mice (PND56) using RNA-seq data from the Brain Atlas resource (58), as the molecular mechanisms of puberty occur primarily in this tissue. As the available data derived from mice at PND56, when Mkrn3 is downregulated (**Figure 2B**), we focused on potential transcriptional repressors. Hinfp factor plays a role in transcriptional repression and DNA methylation through its interaction with the methyl-CpG-binding protein MBD2 (63). In addition, Zbtb7a encodes a transcription factor with repressive activity on various genes through its interaction with the NuRD repressor complex or by recruiting the chromatin regulator HDAC1 (64). A similar function may occur when the *Mkrn3* promoter is hypermethylated and methyl-CPG-binding proteins are recruited with further recruitment of the NuRD repressor complex resulting in Mkrn3 silencing. Finally, putative binding of the negative regulator Hey1, a downstream effector of the Notch signalling pathway (65), was identified in the *Mkrn3* CpG islet region. The Notch pathway plays a key role in the development of the kisspeptin neurons (66), while mutations in genes related to the Notch pathway were found in CPP patients (14, 76). Our *in silico* analysis revealed several transcriptional regulators with binding affinity to the *Mkrn3* CpG islet, which can be used to narrow down target identification in future *in vivo* and *in vitro* experiments. Further experiments are still required to validate the binding and understand the biological significance of these transcription factors in the regulation of Mkrn3 and the subsequent onset of puberty.

Notably, this study did not examine whether changes in methylation levels directly control the molecular mechanism of puberty or are related to the control of other regulatory pathways, but investigated the methylation levels at the *Mkrn3* promoter before, during, and after puberty.

In conclusion, this study determines, during puberty, the methylation status of CpG dinucleotides located in the promoter, the 5’UTR and the first 50 nucleotides of the coding region of the *Mkrn3* gene. It also demonstrates the presence of a CpG islet within the promoter region which is methylated less at the pre-pubertal stage when compared with the pubertal or post-pubertal stage. *In silico* analysis of transcription factor binding sites on the CpG islet showed the recruitment of transcriptional regulators that may affect gene expression. The data presented here will help to further explore the role of methylation in transcriptional regulation of *Mkrn3* at puberty independently of imprinting.

## Data availability statement

The datasets presented in this study can be found in the following online repository. Figshare, Dataset Number: 21443952; DOI: https://doi.org/10.6084/m9.figshare.21443952.v1.

## Ethics statement

The animal study was reviewed and approved by Cyprus Government Veterinary Services (project license CY/EXP/PR.L4/2018).

## Author contributions

PF conceptualized, designed the study, analyzed the data and drafted the manuscript; MM performed the laboratory experiments, analyzed the data and contributed to writing; MaT and KM analyzed the data and proof read the manuscript. MeT, NS and GS reviewed the results and proof read the manuscript. VN conceptualized the study, proof read and revised the manuscript. LP conceptualized the study, reviewed the results and revised the final version of the manuscript. All authors read and approved the final version of the manuscript. All authors contributed to the article and approved the submitted version.
