# Supplementary material for: Methylation status of hypothalamic Mkrn3 promoter across puberty
Source: Front Endocrinol (Lausanne). 2023 Jan 13;13:1075341. doi: 10.3389/fendo.2022.1075341 (PMC9880154; doi:10.3389/fendo.2022.1075341)
Supplement: Supplementary file 1 [file DataSheet_1.docx]

**Supplementary Figure 1**

**
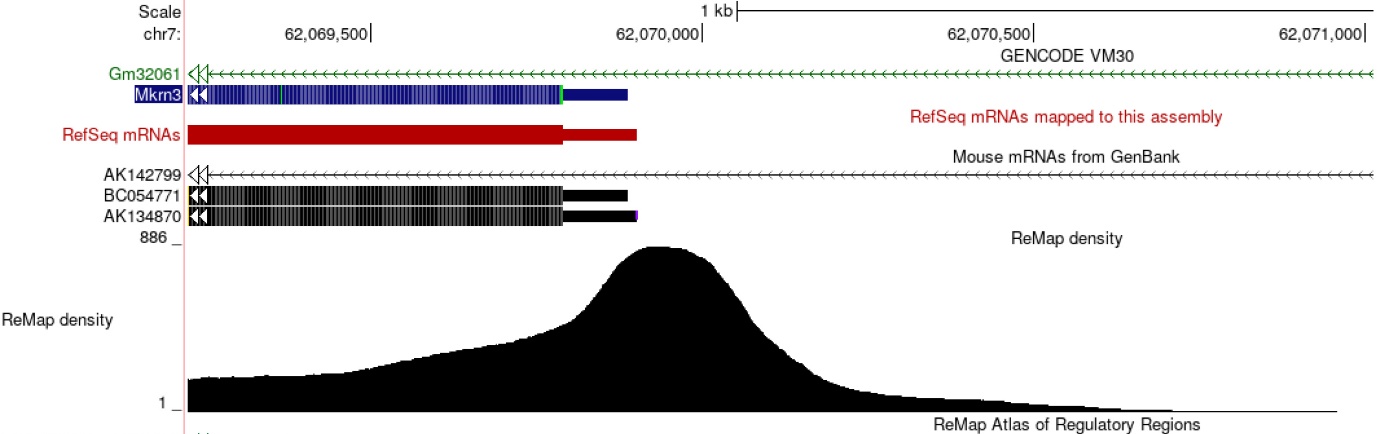
**

**Supplementary Figure 1. Mkrn3 regulatory region on chr7:62,069,224-62,071,878 region.** A ReMap (2022) density at the *Mkrn3* region closed to the TSS as illustrated in UCSC Genome Browser on Mouse (GRCm39/mm39). The dense region upstream of TSS is extended at ~750bp. RefSeq transcript and refseq mRNA for Mkrn3 are depicted with blue and red colour respectively.
